# Novel Gemini ionic liquid for oxidative desulfurization of gas oil

**DOI:** 10.1038/s41598-023-32539-y

**Published:** 2023-04-16

**Authors:** Hoda A. Mohammed, Hamida Y. Mostafa, Dina M. Abd El-Aty, Ashraf M. Ashmawy

**Affiliations:** 1 Refining Department, Egyptian Petroleum Research Institute (EPRI), 1 Ahmed El-Zomor St., Nasr City, Cairo, 11727 Egypt; 2 Analysis and Evaluation Department, Egyptian Petroleum Research Institute (EPRI), 1 Ahmed El- Zomor St., Nasr City, Cairo, 11727 Egypt; 3 Chemistry Department, Faculty of Science, Al-Azhar University, Nasr City, Cairo, 11884 Egypt

**Keywords:** Chemistry, Materials science

## Abstract

The N_1_,N_1_,N_3_,N_3_-tetramethyl –N_1_,N_3_-diphenylpropane-1,3-diaminium dichloride ionic liquid (ILc) is an environmentally friendly catalyst for oxidative–extractive desulfurization of gas oil (sulfur content = 2400 ppm) in the presence of H_2_O_2_ as an oxidizing agent. The precise structure of the prepared IL was confirmed using FT-IR spectroscopy, and^1^H-NMR. The reaction temperature, IL ratios, H_2_O_2_ dosage, and reaction time were studied to assess their effects on the desulfurization efficiency. The thermodynamic parameters of the oxidation reaction were determined. A desulfurization efficiency of 84.7% was obtained after the extractive desulfurization process using acetonitrile as an organic solvent at a solvent to feed ratio of 1:1 (v/v). Furthermore, the prepared IL may be reused for at least six cycles without any significant change in its desulfurization performance or chemical structure, which confirms its high reusability.

## Introduction

Sulfur compounds in fossil fuels present a significant challenge for petroleum refineries^1^. Sulfur oxides (SO_x_) formed during the combustion of sulfur-containing fossil fuels are key contributors to serious air pollution, particularly acid rain and hazy weather^2^**.** Hydrodesulfurization (HDS) is an important process in oil refining. It is commonly used for oil desulfurization, employing metal catalysts to convert organic sulfur in fuels to hydrogen sulfide and related hydrocarbons^3–5^. HDS is widely used in industry to effectively remove sulfides with low boiling points and no steric hindrance such as thioethers and mercaptans^6,7^. However, this technique require large hydrogen consumption, expensive catalysts, and extremely hard reaction conditions^8,9^. Efficient desulfurization can be achieved by multistage extraction desulfurization (EDS)^10,11^; however, the process costs are high because of the high amount of extractant used and the regeneration problems that may occur during the process^1,12,13^. Large amounts of catalysts are required for oxidative desulfurization (ODS)^14–17^. Moreover, regeneration difficulties and poor repeatability are caused by the loss of catalytic active sites during the process. Thus, it is essential to develop new catalysts and extractants with high desulfurization efficiency^18–21^. The oxidation of aromatic sulfides to generate their corresponding sulfones followed by their subsequent removal by extraction in a typical ODS process^15,22–24^. H_2_O_2_ is the most used oxidant in ODS because of its strong reactivity, low cost, and environmental compatibility^25–28^. Flammable and volatile organic solvents are typically used as extractants, which may generate further safety and environmental problems. The development of EDSmethods is constrained by the requirement of a high solvent-to-oil ratio and the lack of environmentally friendly extraction solvents^2,29,30^. Organic solvents can be used as extraction media in EDS; however, they have significant limitations due to their high volatility, low selectivity toward sulfur compounds, and high toxicity^31^. Therefore, new environmentally friendly i.e., biodegradable, nonvolatile, and nontoxic, extraction solvents must be developed. Using ionic liquids (ILs) for EDSis an environmentally friendly method that is increasingly used to remove refractory S-compounds^8^. ILs are salts with low melting points, usually with melting points less than 100 °C. ILs exhibit unique characteristics such as controllable physicochemical characteristics, strong thermal stability, low volatility, and long-term stability. Because of their unique properties, they are used as green solvents for chemical synthesis, fuel desulfurization, and bio-separation^32,33^. Furthermore, ILs have a high ability to form complexes with aromatic sulfur compounds and are immiscible with fuel oils^34^. Zhang et al. 2004^35^ employed l-alkyl-3-methylimidazolium [AMIM] tetrafluoroborate, hexafluorophosphate, and trimethylamine hydrochloride, (TMAC) in (AlCl_3_–TMAC) as ionic liquids. EMIMBF_4_ (E = ethyl), BMIMPF_6_ (B = butyl), BMIMBF_4_, and the heavier AMIMPF_6_ exhibited good selectivity, particularly toward aromatic sulfur and nitrogen compounds, in extractive desulfurization and denitrogenation of transportation fuels. The used ionic liquids are easily regenerated by distillation or water displacement of the absorbed molecules. The aromatic S-containing compounds that were absorbed can be also quantitatively recovered. Organic compounds with a greater aromatic π electron density are absorbed more efficiently. As a result of a steric effect, the alkyl substitution on the aromatic rings significantly decreases the absorption capacity. The size and structure of cations and anion in ILs affect their absorption capacity of aromatic compounds. Without mutual hindrance, the extraction of S- and N-containing compounds can be obtained at low concentrations. Typically, AlCl_3_-TMAC ILs exhibit high absorption capacities for aromatic compounds. To eliminate sulfur compounds from light oils, Lo et al.^36^ used room temperature ILs (RTILs), i.e., 1-butyl-3-methylimidazolium tetrafluoroborate and 1-butyl-3-methylimidazolium hexafluorophosphate, through a combination of solvent extraction and chemical oxidation. In light oils, sulfur compounds can be extracted using RTILs, and the corresponding sulfones can be then produced through S-oxidation (H_2_O_2_-acetic acid) in a one-pot operation. The simultaneous oxidation and extraction of sulfur compounds from light oil increase the desulfurization yield. RTILs can be then reused and recycled without losing their activity.

In this work, N_1_,N_3_-dibenzyl-N_1_,N_1_,N_3_,N_3_-tetramethylpropane-1,3-diaminium chloride was prepared, and its structure was confirmed using several characterization techniques such as Fourier transform infrared spectroscopy (FT-IR) and ^1^H nuclear magnetic resonance (^1^H-NMR). In the ODS of gas oil, the developed IL was used as a catalyst in the occurrence of H_2_O_2_ as the oxidant, and the optimal composition of IL was determined. The optimum conditions of the desulfurization process were obtained by investigating the effects of various operating parameters, including the reaction time, temperature, IL to gas oil volume ratio, and oxidant dosage, on the process. The efficiency of IL toward sulfur removal from gas oil and its recyclability were also investigated, and the thermodynamic parameters of the ODS reaction were determined.

## Experimental methods

### Materials

Benzyl chloride (99%), N,N,N,N-tetramethyl-1,3-propanediamine, and H_2_O_2_ (50 wt%) were obtained from Sigma Aldrich. Ethyl alcohol and acetonitrile (HPLC grade) were obtained from Morgan and Merck chemicals, respectively. All chemicals were of analytical grade and were used directly without further treatments. Gas oil was collected from Cairo oil refining company, Egypt.

### Preparation of the N_1_,N_3_-dibenzyl-N_1_,N_1_,N_3_,N_3_-tetramethylpropane-1,3-diaminium dichloride ILc

**Figure 1:**
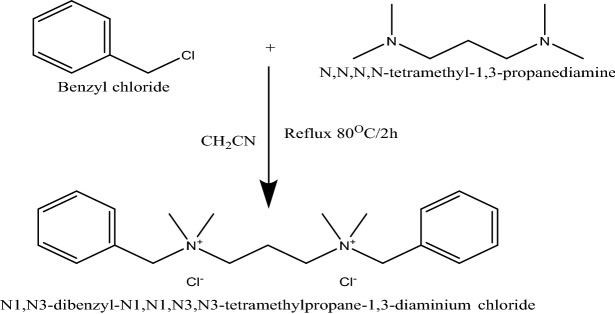
Preparation of the ionic liquid.

N,N,N,N-tetramethyl-1,3-propanediamine (0.01 mol) was dissolved in acetonitrile. Benzyl chloride (0.02 mol) was then added after that the mixture was refluxed at 80 °C for 2 h. Product crystallization was performed 3 times using ethanol with a yield of 80% at a melting point of 70 °C. Figure 1 shows a summary of the preparation process. The structure of the synthesized compound was confirmed using FT-IR spectroscopic analysis with KBr pellets on Perkin Elmer in Egyptian Petroleum Research Institute. ^1^H-NMR spectroscopy was carried out in dimethyl sulfoxide (DMSO) using a Varian Gemini-200 MHz system.

### Desulfurization experiments

At atmospheric pressure, the experiments were conducted in a closed round-bottle flask with a magnetic stirrer and a thermometer. Gas oil (25 ml) was used with different volumes of H_2_O_2_ (5–20 ml) to study their effects on the process. Similarly, the effect of ILc dose of 0.1–0.5 g, a reaction temperature range of 30–80 °C, and a reaction time of 30–240 min on the desulfurization efficiency was investigated. After each treatment, the phase separation was achieved using two layers, an aqueous layer and an oil layer, in a separating funnel^37^, and the treated oil phase was extracted using acetonitrile at a 1:1 (v/v) ratio.

The oil and solvent phases were separated, and the desulfurization efficiency (R) was calculated using Eq. (1).

1 $${\text{R}} = \frac{{{\text{C}}_{{\text{i}}} - {\text{C}}_{{\text{f}}} }}{{{\text{C}}_{{\text{i}}} }} \times 100$$

where C_i_ (ppm) and C_f_ (ppm) are the initial and final sulfur concentrations in the gas oil, respectively.

### Analytical methods

Samples in the upper oil phase were collected for the analysis at different time intervals (30–240 min). A viscometer (Stabinger, Model SVM 3001, Anton Paar) was used for the determination of dynamic viscosity, kinematic viscosity, and density of the samples before and after the treatment according to ASTM D 7042, ASTM D 445, and ASTM D 4052, respectively. The total sulfur concentration of gas oil was determined using a sulfur analyzer according to the standard test method for sulfur in petroleum oil and petroleum products by energy dispersive X-ray fluorescence spectrometry (ASTM D 4294).

**Figure 2:**
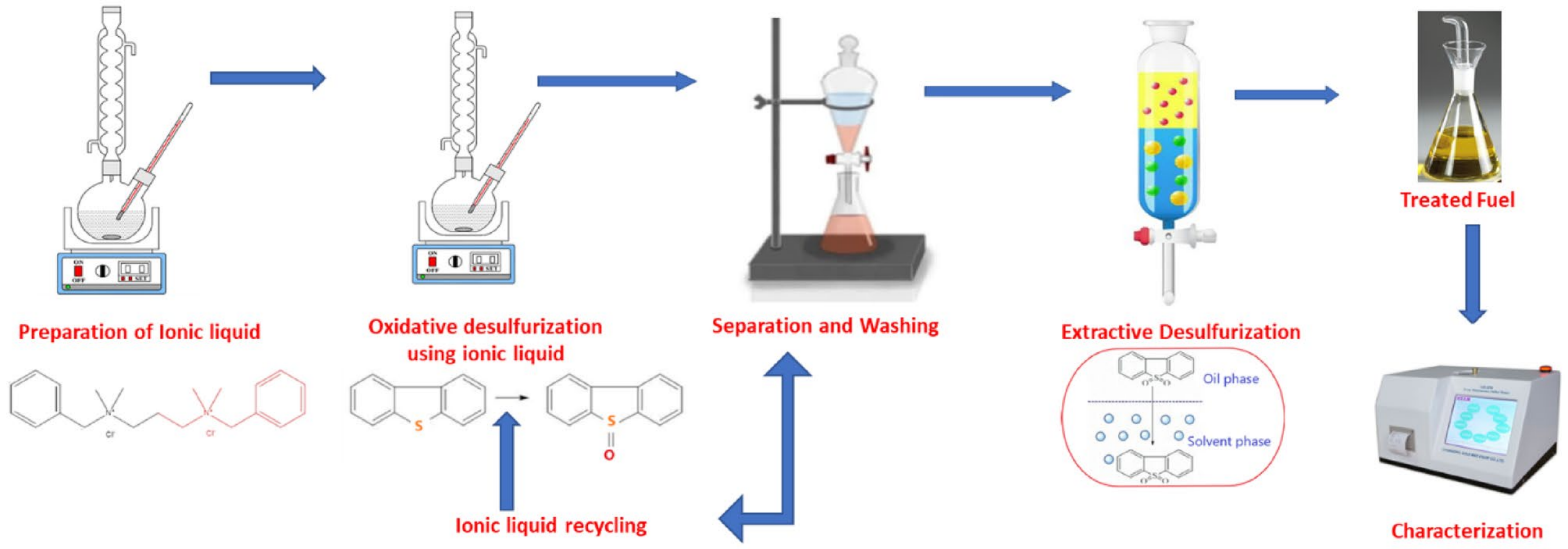
Representation of the experimental steps involved in the preparation of ionic liquid ILc and desulfurization process.

Figure 2 reports the different stages involved in the preparation of ionic liquid ILc and desulfurization process in the present work.

## Results and discussion

The main objective of this study is to remove sulfur compounds, which are usually attached to the aromatic compounds in petroleum fractions, from gas oil by a new IL using an extractive–catalytic ODS process. The physicochemical properties of the gas oil were examined according to the ASTM standard test methods. The results are tabulated in Table 1.

**Table 1:** Physicochemical properties of the gas oil.

Test	Result	Standard test method
Density at 15.56 °C, g/cm^3^	0.8264	ASTM D–4052
Specific gravity at 15.56 °C	0.8272	
API	39.56	
Pour point, °C	− 9	ASTM D–97
Kinematic viscosity at 40 °C, cSt@100 °C	2.6915 15.83	ASTM D–445
Dynamic viscosity at 40 °C, cP@100 °C	2.1773 15.83	ASTM D–7042
Sulfur content, ppm	2400	ASTM D–4294
Color	1	ASTM D–1500
Ash content, wt%	Nil	ASTM D–482
Carbon residue, wt%	Nil	ASTM D–524
Flash point, °C	80	ASTM D–93
Aniline point, °C	78	ASTM D–611
Calorific value, mJ/kg	462,554	ASTM D–240

### Confirmation of the ILc structure

#### FT-IR

**Figure 3:**
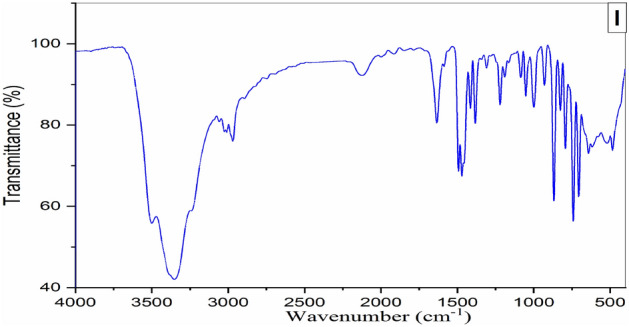
FT-IR spectra of the prepared ILc.

The infrared spectra of the purified ILc prepared in this study are presented in Fig. 3. The characteristic FT-IR bands of Fig. 3 are listed in Table 2. We noted that for ILc band appear at 3001 and 3050 could be due to Aromatic (–CH–) groups. The peak at about 2969 cm^−1^ due to Aliphatic (–CH–) groups. The peak at 1634 cm^−1^ is assigned to the stretching vibration of C=C Aromatic. In addition to peak appear at 1220 cm^−1^ is assigned to C–N. Finally, the FT-IR analysis indicates the presence of the IR bands that related to the chemical structures of new ILc.

**Table 2:** FT-IR bands of N_1_,N_1_,N_3_,N_3_-tetramethyl -N1,N3-diphenylpropane-1,3-diaminium dichloride.

Cpd	CH–H_2_O	Aromatic C–H	Aliphatic C–H	Aromatic C=C	C–N
IL	3450	3001–3050	2969	1634	1220

*N.B:* The bands observed at 3450 cm^−1^ were assigned to the stretching vibrations bands of the hydrogen-bonded H_2_O molecules^38^.

#### ^1^H-NMR

**Figure 4:**
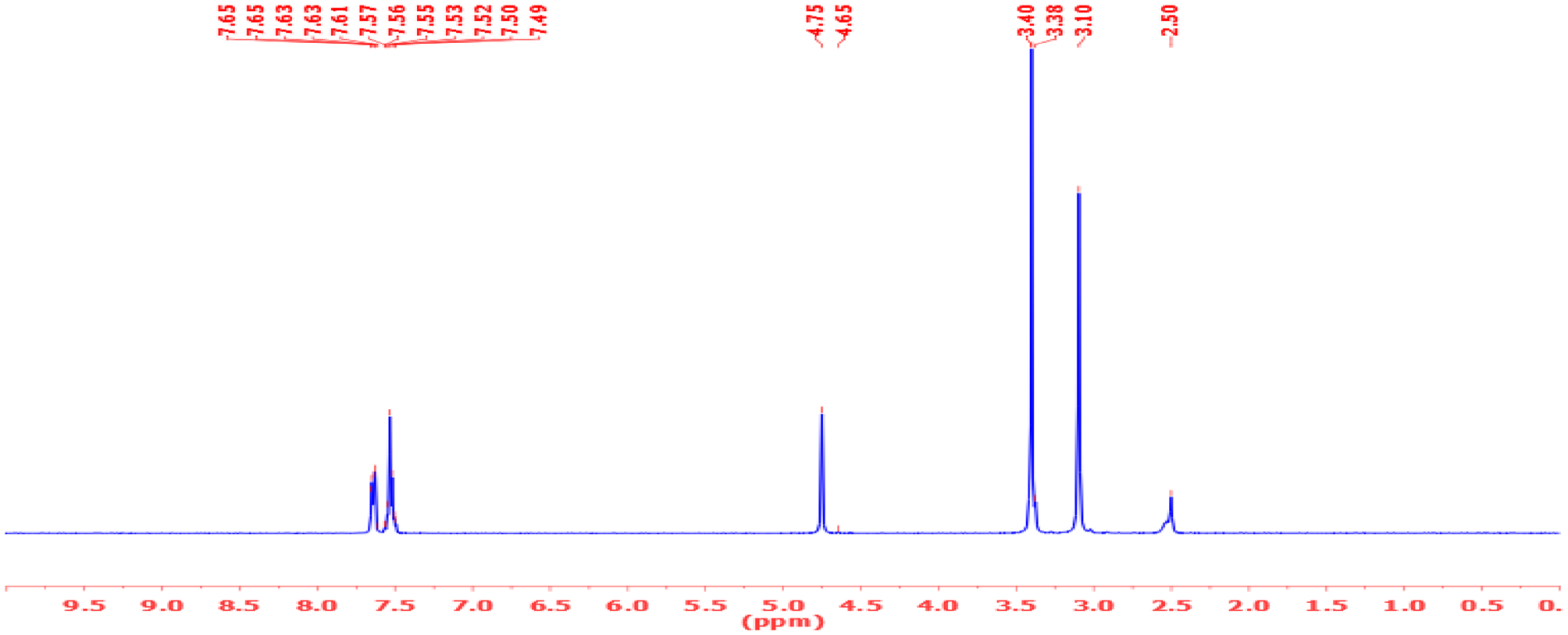
^1^H-NMR spectra of the prepared ILc.

The chemical structure of the novel investigated ILc was determined by ^1^H NMR spectroscopy. The detailed spectra shown in Fig. 4 agreed with the designed structure. Table 3 lists the changes in chemical shifts for several sorts of protons in the novel investigated ILc. Aromatic protons, (A, B and C), appeared at (9.07, 7.81 and 7.73) respectively. Aliphatic proton (D, E, F and G) appears at (4.75, 3.40, 3.38 and 3.10). No impurities were observable in ^1^H spectra.

**Table 3:** Chemical shifts in the prepared IL.


Chemical shifts of different types of protons (ppm)							
Cpd	A	B	C	D	E	F	G
	7.65 (t)	7.57 (t)	7.52 (d)	4.75 (s)	3.40 (s)	3.38 (t)	3.10 (t)

*N.B.*: s: singlet, d: doublet, and t: triplet.

### Effect of the reaction temperature

**Figure 5:**
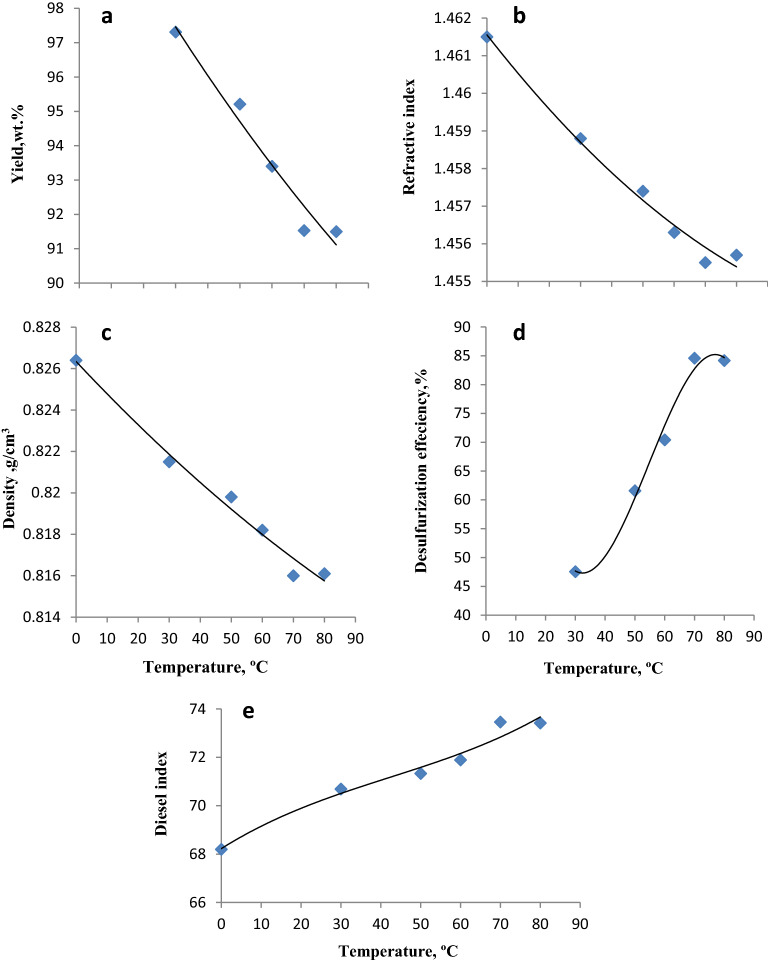
Effect of temperature on yield, density, refractive index, desulfurization efficiency, and diesel index of gas oil treated with the ILc.

The impact of temperature on the desulfurization efficiency is a critical factor in defining the potential of the synthesized ILc. Typically, increasing the temperature accelerates the decomposition of H_2_O_2_ in water to generate nascent oxygen atoms, or (OH) ions, which oxidize sulfur compounds to varying degrees. To investigate the effect of the reaction temperature on the desulfurization efficiency, experiments were carried out at 30 °C, 50 °C, 60 °C, 70 °C, and 80 °C (Fig. 5 and Table S1). The increase in the temperature from 30 to 80 °C was accompanied by a decrease in the yield from 97.31 to 91.5 wt%, respectively (Fig. 5a). Density and refractive indices decreased (Fig. 5b,c)^39^**,** and on the other hand diesel indices increased because of the increase of the aromatic recoveries (Fig. 5e). The aromatic and sulfur recoveries exhibited different degrees of increase. At an operation temperature of 30 °C, the desulfurization efficiency could only reach 47.54%, which indicates that the ILc could not catalyze the reaction efficiently at a relatively low temperature. However, when the temperature increased to 50 °C, 60 °C, and 70 °C, the desulfurization efficiency remarkably increased to 61.58%, 70.42%, and 84.58%, respectively, (Fig. 5d). Sulfur removal was restricted by the unproductive decomposition of H_2_O_2_ at elevated temperatures. The loss of H_2_O_2_ due to its decomposition to H_2_O and O_2_ at high temperatures^40^ may restrict the effective formation of active oxidation species. Similarly, the H_2_O impurity generated by the thermal decomposition of H_2_O_2_ hindered the catalytic activity of ILc, leading to a reduction in the desulfurization efficiency^41^. Therefore, the desulfurization efficiency did not significantly improve when the temperature increased from 70 to 80 °C. Thus, the optimal reaction temperature was selected to be 70 °C.

### Effect of reaction time

**Figure 6:**
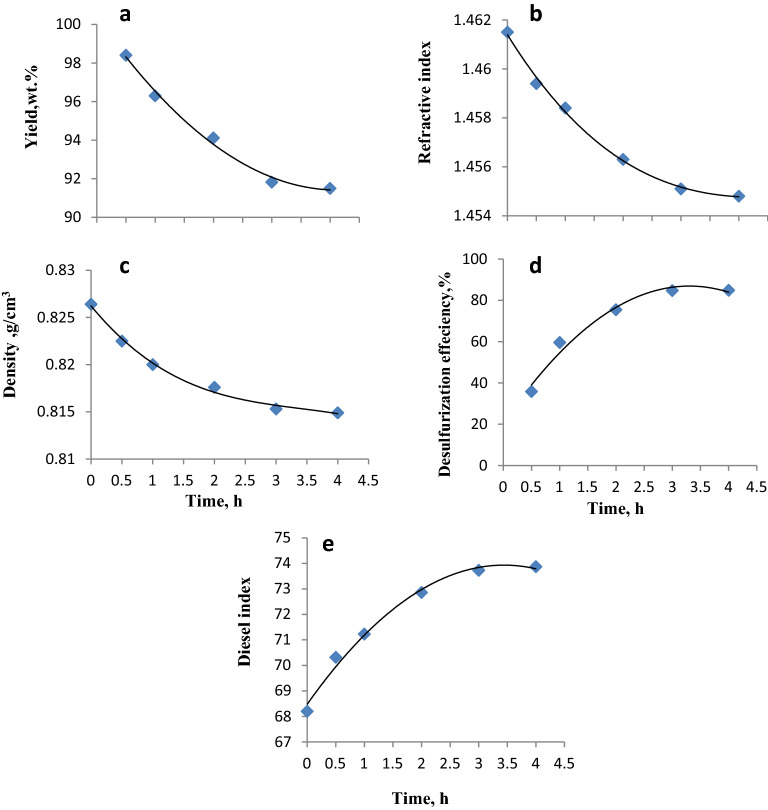
Effect of the reaction time on the yield, density, refractive index, desulfurization efficiency, and diesel index of gas oil treated with the ILc.

The selectivity of the oxidation process determines the utility of the desulfurization system. The sulfur compounds should be rapidly and selectively oxidized. Thus, the oxidation time of the gas oil was determined. To determine the minimum reaction time for complete desulfurization of sulfur compounds at 70 °C, the performance of the ODS process using ILc was assessed at different times (0.5, 1, 2, 3, and 4 h). Figure 6 and Table S2 show the desulfurization efficiencies and their associated species yields, densities, refractive indices, and diesel indices of the oxidized gas oil. H_2_O_2_ (10 ml) and ILc (0.5 g) were used for this test at an oxidation temperature of 70 °C, which is the optimal temperature selected in the previous step. The results presented in Fig. 6 indicate that the increase in the reaction time from 0.5 to 3 h was associated with a continuous sharp decrease in the yield, refractive indices, and density (Fig. 6a–c). This was accompanied by a continuous increase in the desulfurization efficiency and diesel indices (Fig. 6d,e). This can be attributed to the continuous increase in the reaction time, which can increase the π–π interaction between the ILc molecules and sulfur compounds in the reaction media. This enhanced the extraction of the sulfur compounds and enable the oxidizing agent (H_2_O_2_) to produce the prior species in the media to complete the ODS process. Further elongation of the reaction time to 4 h showed no significant increase in the desulfurization efficiency or the associated characteristics. Therefore, 3 h was considered a suitable reaction time.

### Effect of the ILc dosage

**Figure 7:**
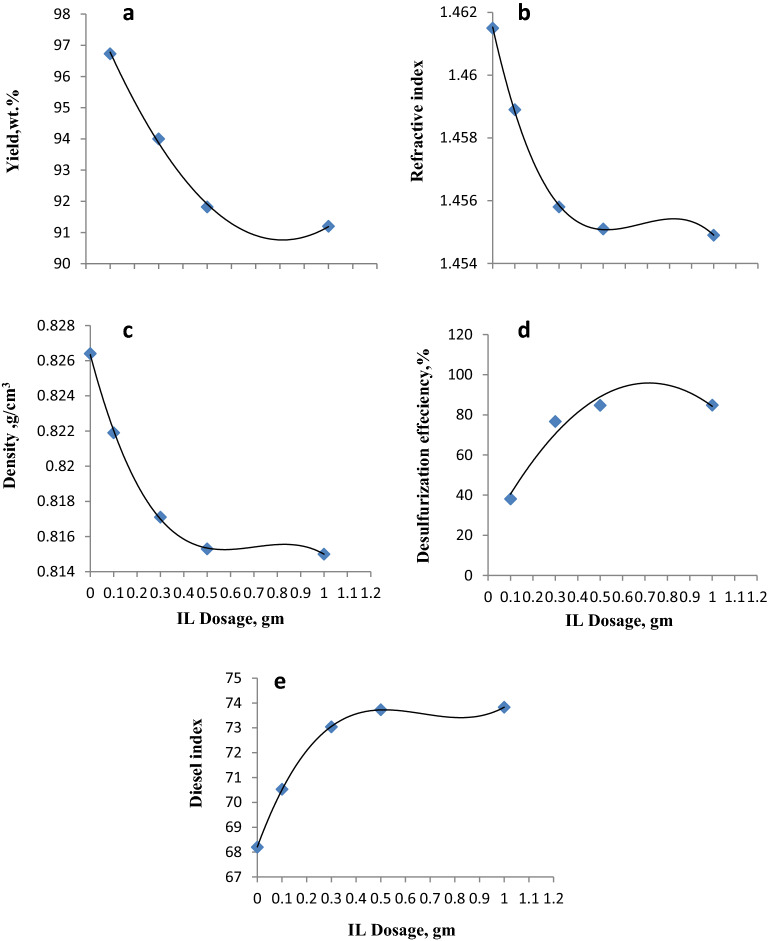
Effect of the ILc dosage on the yield, density, refractive index, desulfurization efficiency, and diesel index of gas oil treated with the ILc.

Figure 7 and Table S3 show the effect of the ILc dosage on the process of extractive–ODS process and its subsequent relevant changes in the yield, physical characteristics, desulfurization efficiency, and diesel indices of the produced gas oil. Four doses of ILc (0.1, 0.3, 0.5, and 1 g) were used during this step. The optimal conditions, i.e., reaction temperature = 70 °C and reaction time = 3 h, selected in the previous steps were employed in the current test using H_2_O_2_ (10 ml). The increase in the ILc dosage can be an efficient strategy to improve the desulfurization efficiency. The desulfurization efficiency significantly increased from 70.53 to 73.73% (Fig. 7d) when the ILc dosage increased from 0.1 to 0.5 g. Because ILc was used as both a catalyst and an extractant, the increase in the ILc dosage could enhance the extraction efficiency and increase the catalytic active sites, which significantly improve sulfur removal. Nevertheless, no significant differences were observed in the characteristics of the produced gas oil at higher ILc dosage (1 g). That is, nearly the same percentages of desulfurization efficiency were observed. Thus, a lower ILc dosage (0.5 g) was considered the optimal dosage for industrial applications from an economic point of view.

### Effect of the H_2_O_2_ dose

**Figure 8:**
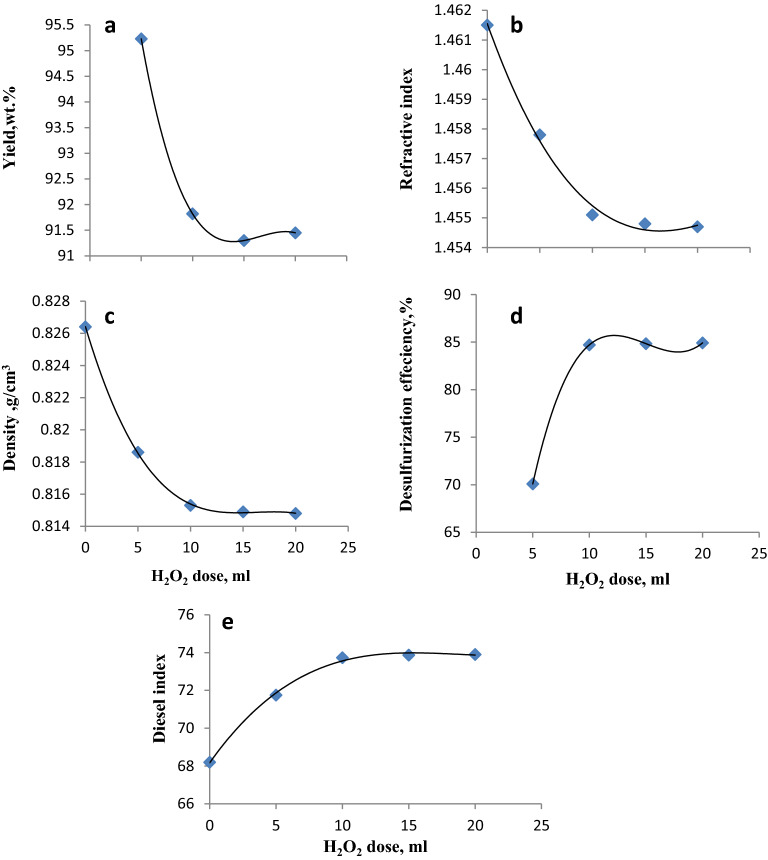
Effect of the H_2_O_2_ dosage on the yield, density, refractive index, desulfurization efficiency, and diesel index of gas oil with ILc.

H_2_O_2_ is a potent, environmentally friendly, and inexpensive oxidizing agent. The impact of using H_2_O_2_ was also tested (Fig. 8 and Table S4). The experiments in this stage were carried out under the optimal conditions selected above (ILc dosage = 0.5 g, reaction time = 3 h, and oxidation temperature = 70 °C). Four H_2_O_2_ dosages (5, 10, 15, and 20 ml) were evaluated in this step. Figure 8 indicates that when the oxidizing agent dose increased from 5 to 10 ml, the yield (Fig. 8a) significantly decreased, showing a clear improvement in the refractive indices and density (Fig. 8b,c). This was accompanied by a simultaneous increase in the desulfurization efficiency (Fig. 8d) and diesel indices (Fig. 8e) until approximately fixed values were detected when the H_2_O_2_ dose increased from 10 to 15 then to 20 ml. This verifies that the excessive use of H_2_O_2_ is not desirable because it results in the dilution of ILc and increases the cost. Therefore, a H_2_O_2_ dose of 10 ml was chosen as the optimal dose in this study.

### Thermodynamics of the oxidative desulfurization of diesel fuel

The thermodynamic parameters of the ODS reaction were estimated as follow:

The standard entropy change (ΔS°, J/(mol K)), standard enthalpy change (ΔH°, kJ/mol), and standard free energy change (ΔG°, kJ/mol)^42^ were calculated using Eqs. (2) and (3).

2 $$Ln\;Kd = \frac{{\Delta S^{\circ} }}{R} - \frac{{\Delta H^{\circ} }}{RT}$$

3 $$\Delta G^{\circ} = \Delta H^{\circ} - T\Delta S^{\circ}$$

where T is the absolute temperature (K), Kd is the distribution coefficient (L/g), and R is the perfect gas constant = 8.314 J/mol K.

**Figure 9:**
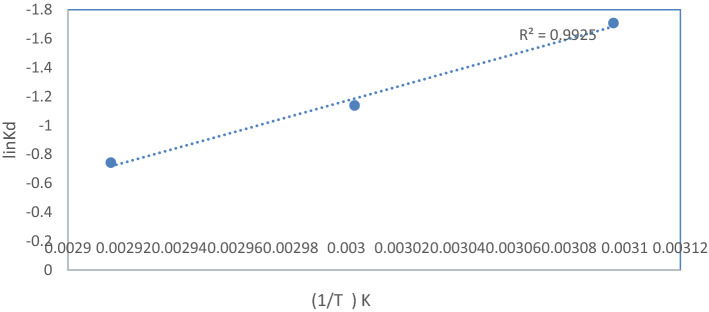
Thermodynamic behavior of the oxidative desulfurization reaction.

The thermodynamic parameters of the reaction can be calculated using Eqs. ((2) and (3)), the results are listed in Table 4 and Fig. 9 show thermodynamic behavior of the oxidative desulfurization reaction.

**Table 4:** Thermodynamic parameters of the oxidative desulfurization reaction.

ΔH° (kJ/mol)	ΔS° [J/(mol K)]	ΔG° at different T (kJ/mol)		
31	0.08	303 K	333 K	353 K
		4.5	1.8	0.09

As shown in Table 4, the values of ΔH°, ΔS°, and ΔG° are positive, suggesting that the reaction is endothermic with increase in the temperature. Thus, the efficiency of the oxidation and randomness of the reaction increased, and the reaction was nonspontaneous. The low values of the standard free energy mean that the reaction can proceed easily^43^.

### Recycling of the ILc

From environmental and economic standpoints, ILc regeneration and recycling are vital processes. Repeated trials of eliminating sulfur compounds from heavy gas oil under optimal conditions were conducted to verify the recycling performance of ILc. A biphasic system was observed in the reactor after repeating each cycle. The upper phase was carefully removed from the system by decanting, and the ILc phase was dried under vacuum to remove any remaining water and H_2_O_2_. The reactor was then charged with fresh gas oil and H_2_O_2_ for the next cycle.

**Figure 10:**
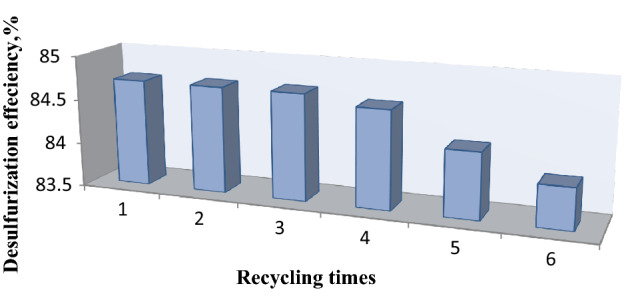
Effect of recycle times of the prepared ILc on the desulfurization efficiency.

**Figure 11:**
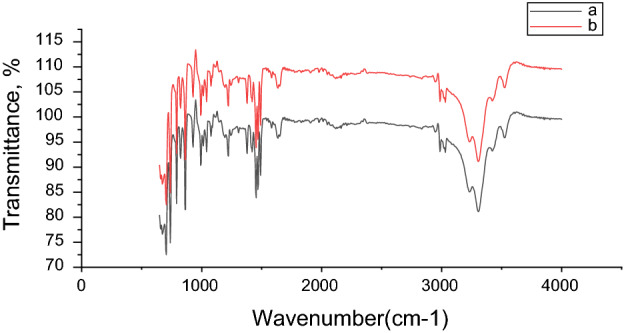
FT-IR analysis of the (**a**) fresh ILc and (**b**) recycled ILc.

After six repeated cycles, the desulfurization efficiency only dropped from 84.71 to 83.97% (Fig. 10). This slight drop could be attributed to one of two factors. First, trace losses of ILc during the separation and drying processes are unavoidable. Second, the oxidative products of the sulfur compounds concentrated in the system and coated the surface of ILc, reducing its phase transfer capabilities. Nonetheless, based on these results, ILc shows good recyclability through a low-cost regeneration approach and may be suitable for industrial applications. In addition, FT-IR analyses of both fresh and recycled ILc (Fig. 11) were performed to investigate the stability of ILc during the desulfurization process. There was no discernible difference between before and after the reactions.

### Desulfurization mechanism

**Figure 12:**
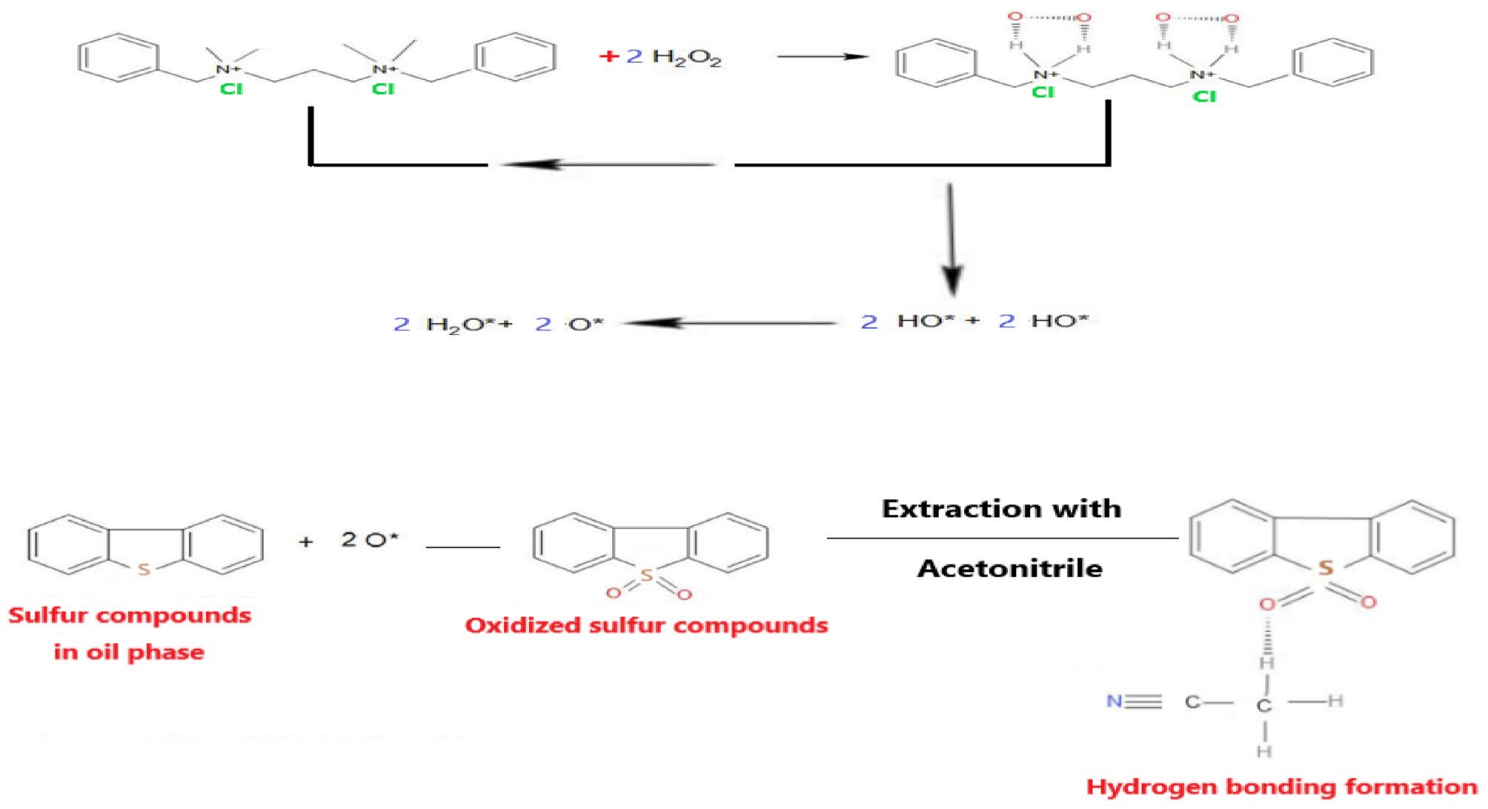
Desulfurization mechanism.

Gas oil is desulfurized through a very effective and selective process using the developed Gimini ionic liquid ILc as a catalyst (Fig. 12). That the ILc contains two positive active sites, which combine with two molecules of hydrogen peroxide and produce active species, which perform their beneficial role in completing the oxidation process. The effect of ILc increases the rate of decomposition of H_2_O_2_ to 2OH*, which is unstable and undergo O–H bond break to generate H_2_O* and O*, which are the most active species for an ODS reaction because of their low activation energy. The effectiveness of the desulfurization process is significantly impacted by the π–π interactions between the aromatic sulfur compounds and aromatic ILc^44^.

Our Gemini ionic liquid ILc performs more catalytically than its mono-cationic version and this difference can be related to the fact that Gemini ionic liquids have two basic sites, whilst mono-cationic ionic liquids only have one. Gemini ionic liquids have two basic sites that are close to one another and work well in tandem to increase the catalytic efficiency. As Gemini ionic liquids have stronger basic sites than mono-cationic ILc, oxidation process proceed more quickly. They display greater stability and more potent alkalinity because of their more powerful and concentrated basic sites.

In Table 5, the performance of the novel introduced N1,N1,N3,N3-tetramethyl-N1,N3-diphenylpropane-1,3-diaminium dichloride was compared with certain previously referred ILs for further studs.

**Table 5:** Comparison between the N1,N1,N3,N3-tetramethyl-N1,N3-diphenylpropane-1,3-diaminium dichloride ILc and other ILs used in ODS.

IL	Fuel (model or real)	Conditions				Desulfurization effeciency%	Ref.
		Temp., °C	Time,h	Oil:IL	H_2_O_2_:Oil		
[HNMP][H_2_PO_4_]	Diesel; DBT 2000 ppm	60	5	1:1	16:1	64.3	^45^
[BMIM]Cl/FeCl_3_	Diesel; DBT 1000 ppm	30	0.5	1.5:1	20:1	71.3	^46^
[Hnmp][HCOO]	Diesel; 1000 ppm	40	1.5	1:1	10:1	80	^47^
[hnmp]HSO_4_	Gasoline fuel; 260 ppm	60	4	1:1	16:1	57	^48^
([BPy][BF_4_]	Diesel; DBT; 1000 ppm	30	0.67	1:1	18:1 72:1	81.2 89.5	^7^
N1,N1,N3,N3-tetramethyl -N1,N3-diphenylpropane-1,3-diaminium dichloride	Gas oil; S. comp. 2400 ppm	70	3	1:0.02	0.4:1	84.7	This work

In addition to the declared factors, the type of the catalyst itself would be effective as well. In Table 5, the novel ILc is contrasted with existing types of ionic liquids employed in the same application—oxidative desulfurization process—to show off its excellent catalytic performance. As a consequence, Gemini ionic liquid ILc performed superior than the other ionic liquid types described in terms of both results and efficiency. So, for the oxidative desulfurization process, the ILc with the best and most competitive performance (84.7%) for real gas oil (2400 ppm sulphur content) is chosen. This is due to the using of a very little amount of the catalyst ratio—oil:ILc = 1:0.02—and the ILc helped on a huge reduction in the amount of the oxidizing agent—H_2_O_2_:Oil = 0.4:1—which makes the process very cost-effective when applied in the industrial field.

## Conclusion

In this study a novel Gemini IL (N1,N1,N3,N3-tetramethyl-N1,N3-diphenylpropane-1,3-diaminium dichloride) was successfully synthesized and characterized using ^1^H-NMR and FT-IR spectroscopy. It was then used as a catalyst in ODS of real gas oil with a sulfur content of 2400 ppm. The N1,N1,N3,N3-tetramethyl-N1,N3-diphenylpropane-1,3-diaminium dichloride IL exhibited a high desulfurization efficiency of 84.7% under optimal conditions (H_2_O_2_:Oil = 0.4:1, IL:Oil = 0.02:1, reaction temperature = 70 °C, and reaction time = 3 h). Based on the thermodynamic analysis of the ODS process, the values of ΔH° indicated that the reaction is endothermic with increase the temperature. The IL can be directly reused, and they exhibited good recyclability for six times.

## Supplementary Information

Supplementary Tables.

## Data Availability

All data generated or analysed during this study are included in this published article (and its supplementary information files).
