# Supplementary material for: Novel Gemini ionic liquid for oxidative desulfurization of gas oil
Source: Sci Rep. 2023 Apr 16;13:6198. doi: 10.1038/s41598-023-32539-y (PMC10106454; doi:10.1038/s41598-023-32539-y)
Supplement: Supplementary file 1 — Supplementary Tables. [file 41598_2023_32539_MOESM1_ESM.docx]

**Novel Gemini Ionic Liquid for Oxidative Desulfurization of Gas Oil**

Hoda A. Mohamed^2^, Hamida Y. Mostafa^1*^, Dina M. Abd El-Aty^1^, Ashraf M. Ashmawy^3^

^1^ Refining Department, Egyptian Petroleum Research Institute (EPRI), 1 Ahmed El- Zomor St., Nasr City, 11727, Cairo, Egypt

^2^  Analysis and Evaluation Department, Egyptian Petroleum Research Institute (EPRI), 1 Ahmed El- Zomor St., Nasr City, 11727, Cairo, Egypt

^3^ Chemistry Department, Faculty of Science, Al-Azhar University, Nasr City, Cairo, 11884, Egypt

-------------------------------------------

* Corresponding author. E-mail: H.Y.Mostafa@epri.sci.eg (Hamida Y. Mostafa)

**Table (S1) Effect of Temperature on physical characterization, desulfurization efficiency, and diesel index of gas oil treated with the IL:**

| **Temp, °C** | **Refractive index** | **Density ,g/cm^3^**  **at 15.56 °C** | **API** | **Sulfur content,ppm** | **Desulfurization efficiency,%** | **Kin.viscosity**  **at 40 °C** | **Dyn.viscosity**  **at 40 °C** | **Aniline point, °C** | **Diesel index** |
| --- | --- | --- | --- | --- | --- | --- | --- | --- | --- |
| Feed | 1.4615 | 0.8264 | 39.56 | 2400 | --- | 2.6915 | 2.1773 | 78 | 68.20 |
| 30 | 1.4588 | 0.8215 | 40.58 | 1259 | 47.54167 | 2.6669 | 2.1434 | 79 | 70.69 |
| 50 | 1.4574 | 0.8198 | 40.94 | 922 | 61.58333 | 2.5734 | 2.064 | 79 | 71.33 |
| 60 | 1.4563 | 0.8182 | 41.27 | 710 | 70.41667 | 2.5271 | 2.022 | 79 | 71.89 |
| 70 | 1.4555 | 0.816 | 41.74 | 370 | 84.58333 | 2.5027 | 1.9973 | 80 | 73.46 |
| 80 | 1.4557 | 0.8161 | 41.72 | 380 | 84.16667 | 2.5088 | 2.002 | 80 | 73.42 |

**Table (S2): Effect of Time on physical characterization, desulfurization efficiency, and diesel index of gas oil treated with the IL:**

| **Time, h** | **Refractive index** | **Density ,g/cm^3^**  **at 15.56 °C** | **API** | **Sulfur content,ppm** | **Desulfurization efficiency,%** | **Kin.viscosity**  **at 40 °C** | **Dyn.viscosity**  **at 40 °C** | **Aniline point, °C** | **Diesel index** |
| --- | --- | --- | --- | --- | --- | --- | --- | --- | --- |
| 0 | 1.4615 | 0.8264 | 39.56 | 2400 | --- | 2.6915 | 2.1773 | 78 | 68.20 |
| 0.5 | 1.4594 | 0.8225 | 40.37 | 1540 | 35.83 | 2.6731 | 2.1516 | 79 | 70.32 |
| 1 | 1.4584 | 0.82 | 40.89 | 970 | 59.58 | 2.5791 | 2.069 | 79 | 71.23 |
| 2 | 1.4563 | 0.8176 | 41.40 | 590 | 75.42 | 2.5197 | 2.015 | 80 | 72.86 |
| 3 | 1.4551 | 0.8153 | 41.89 | 367 | 84.71 | 2.5024 | 1.9954 | 80 | 73.73 |
| 4 | 1.4548 | 0.8149 | 41.97 | 363 | 84.88 | 2.5019 | 1.9938 | 80 | 73.87 |

**Table (S3) Effect of Ionic Liquid Dosage on physical characterization, desulfurization efficiency, and diesel index of gas oil treated with the IL:**

| **IL, g** | **Refractive index** | **Density ,g/cm3**  **at 15.56 °C** | **API** | **Sulfur content,ppm** | **Desulfurization efficiency,%** | **Kin.viscosity**  **at 40 °C** | **Dyn.viscosity**  **at 40 °C** | **Aniline point, °C** | **Diesel index** |
| --- | --- | --- | --- | --- | --- | --- | --- | --- | --- |
| 0 | 1.4615 | 0.8264 | 39.56 | 2400 | --- | 2.6915 | 2.1773 | 78 | 68.20 |
| 0.1 | 1.4589 | 0.8219 | 40.49 | 1485 | 38.13 | 2.6700 | 2.1467 | 79 | 70.53 |
| 0.3 | 1.4558 | 0.8171 | 41.50 | 560 | 76.67 | 2.5182 | 2.012 | 80 | 73.04 |
| 0.5 | 1.4551 | 0.8153 | 41.89 | 367 | 84.71 | 2.5024 | 1.9954 | 80 | 73.73 |
| 1 | 1.4549 | 0.815 | 41.95 | 365 | 84.79 | 2.5021 | 1.9944 | 80 | 73.83 |

| **H_2_O_2_, ml** | **Refractive index** | **Density ,g/cm^3^**  **at 15.56 °C** | **API** | **Sulfur content,ppm** | **Desulfurization efficiency,%** | **kin.viscosity**  **at 40 °C** | **Dyn.viscosity**  **at 40 °C** | **Aniline point,°C** | **Diesel index** |
| --- | --- | --- | --- | --- | --- | --- | --- | --- | --- |
| 0 | 1.4615 | 0.8264 | 39.56 | 2400 | --- | 2.6915 | 2.1773 | 78 | 68.20 |
| 5 | 1.4578 | 0.8186 | 41.19 | 718 | 70.08 | 2.5302 | 2.026 | 79 | 71.75 |
| 10 | 1.4551 | 0.8153 | 41.89 | 367 | 84.71 | 2.5024 | 1.9954 | 80 | 73.73 |
| 15 | 1.4548 | 0.8149 | 41.97 | 364 | 84.83 | 2.5020 | 1.9938 | 80 | 73.87 |
| 20 | 1.4547 | 0.8148 | 41.99 | 362 | 84.92 | 2.5008 | 1.9926 | 80 | 73.90 |

**Table (S4) Effect of H_2_O_2_ Dose on physical characterization, desulfurization efficiency, and diesel index of gas oil treated with the IL:**
